# Using co‐design methods to develop new personalised support for people living with Long Covid: The ‘LISTEN’ intervention

**DOI:** 10.1111/hex.14093

**Published:** 2024-05-24

**Authors:** Fiona Jones, Anne Domeny, Jessica Fish, Fiona Leggat, Ian Patel, Jackie McRae, Carol Rowe, Monica E. Busse

**Affiliations:** ^1^ Population Health Research Institute St George's University of London London UK; ^2^ Bridges Self‐Management London UK; ^3^ LISTEN Lived Experience Advisory Group London UK; ^4^ Department of Clinical Neuropsychology and Clinical Health Psychology St George's University Hospitals NHS Foundation Trust London UK; ^5^ Mental Health and Wellbeing, School of Health and Wellbeing University of Glasgow Glasgow UK; ^6^ Centre for Allied Health, Institute for Medical, Biomedical and Allied Education St George's University of London London UK; ^7^ Centre For Trials Research, School of Medicine Cardiff University Cardiff Wales UK

**Keywords:** co‐design, fidelity, Long Covid, personalised, self‐management support, training

## Abstract

**Introduction:**

Many Covid‐19 survivors are living with unresolved, relapsing and remitting symptoms and no ‘one size’ of treatment is likely to be effective for everyone. Supported self‐management for the varied symptoms of Long Covid (LC) is recommended by the National Institute for Health and Care Excellence in the United Kingdom. We aimed to develop a new personalised support intervention for people living with LC using a structured co‐design framework to guide replication and evaluation.

**Methods:**

We used the improvement methodology, Experience‐Based Co‐Design, in an accelerated form to harness the collective experiences of people with LC. Incorporating evidence from ‘Bridges Self‐Management’ (Bridges) an approach in which healthcare professionals (HCPs)are trained to support knowledge, confidence and skills of individuals living with long term conditions. Co‐designed resources are also central to Bridges. Adults who self‐identified as living with or recovered from LC, from England or Wales, aged 18 years and over were recruited, and HCPs, with experience of supporting people with LC. Participants took part in a series of small co‐design group meetings and larger mixed meetings to agree priorities, core principles and generate resources and intervention content.

**Results:**

People with LC (*n* = 28), and HCPs (*n* = 9) supported co‐design of a book (hard‐copy and digital form) to be used in 1:1 support sessions with a trained HCP. Co‐design stages prioritised stories about physical symptoms first, and psychological and social challenges which followed, nonlinear journeys and reconceptualising stability as progress, rich descriptions of strategies and links to reputable advice and support for navigating healthcare services. Co‐design enabled formulation of eight core intervention principles which underpinned the training and language used by HCPs and fidelity assessments.

**Conclusion:**

We have developed a new personalised support intervention, with core principles to be used in one‐to‐one sessions delivered by trained HCPs, with a new co‐designed book as a prompt to build personalised strategies and plans using narratives, ideas, and solutions from other people with LC. Effectiveness and cost effectiveness of the ‘LISTEN’ intervention will be evaluated in a randomised controlled trial set within the context of the updated Framework for Developing and Evaluating Complex Interventions.

**Patient and Public Contribution:**

The LISTEN Public and Patient Involvement (PPI) group comprised seven people living with LC. They all contributed to the design of this study and five members were part of a larger co‐design community described in this paper. They have contributed to this paper by interpreting stages of intervention design and analysis of results. Three members of our PPI group are co‐authors of this paper.

## 1 INTRODUCTION

The condition of ‘Long Covid’, the name given by a community of people experiencing long‐lasting symptoms following Covid‐19 illness, combines the National Institute of Health and Care Excellence (NICE) terms of ‘ongoing symptomatic covid‐19’ (symptoms for 4–12 weeks) and post‐covid syndrome (symptoms beyond 12 weeks).^1^ ^2^ In the United Kingdom, estimates state at least 1.9 million people meet the criteria for Long Covid (LC) and of these 1.3 million have symptoms lasting for more than a year and 762,000 symptoms lasting more than 2 years.^3^ While the full extent is unknown, symptoms consistent with LC can occur across all body systems, and over 200 symptoms have been identified.^4^ Clusters of symptoms are now recognised dominated by fatigue, breathlessness, heart palpitations, muscle and joint pain and cognitive dysfunction.^5^ In 2023, Davis et al. published a review that outlined the similarities between LC and other viral‐onset conditions but recognised that the understanding of aetiology and progression of LC is at an early stage with limited mechanistic studies. Risks such as socioeconomic factors and inability to rest in the early stages of the infection, female sex, type 2 diabetes, Epstein–Barr virus reactivation and other existing disorders have helped to advance theories about possible underlying causes and triggers. However, these hypotheses do not explain the third of people with LC that have no identified pre‐existing conditions.^6^ Numerous manifestations of symptoms inevitably lead to difficulties with everyday activities, work, family life and reduced quality of life.^4^

Knowledge about how and when people with LC gain access to health services and the level and quality of care they receive is also vital to fully understand the impact on everyday life. In 2022, the STIMULATE‐ICP Delphi study in England explored pathways to care and experiences of seeking treatment, treatment offered and referral to specialist support amongst patients, general practitioners (GPs) and healthcare professional (HCPs) with experience of LC and other long‐term conditions (LTCs).^7^ It was found that only 51% reported that the HCP was able to recognise their problem when explicitly asked, lower than for other LTCs. Even when referrals were made to specialist services, there were significant delays and waiting lists were high. There were also discrepancies between patients' perceived significance of their problems and the levels of actions taken, and having access did not necessarily mean the HCPs were equipped and motivated to provide adequate support.^7^ Qualitative interviews with a subset of STIMULATE‐ICP Delphi respondents confirmed the role played by peer support and online communities in navigating access to LC services and the crucial role of self‐advocacy.^8^ Other studies have highlighted a myriad self‐generated strategies necessary to manage day‐to‐day life with LC.^9^ These studies highlight the additional burden of ‘needing to be believed’ as opposed to the positive impact of a trusting and credible relationship between HCPs and patients ^10^ As those living with LC experience such varied and complex relapsing and remitting symptoms, pathways of care that rely on generic approaches that lack individual adaptation are unlikely to be fully effective.^11^ A longitudinal qualitative study in the United Kingdom found while access to specialist services and ‘being in the system’ was appreciated by people with LC this could also restrict chances to access ongoing holistic and integrated care, and a lack of understanding about complexity of symptoms from HCPs still exists.^12^

Critically applying treatments without the understanding of effect in LC and personalising to each individual could also be potentially harmful. An editorial published in 2022^13^ drew on existing clinical and lived experience, and outputs from debates and consensus from an international forum of physiotherapists, including those living with LC and their allies from research, peer support, education and advocacy. Their recommendations for ‘Safe Rehabilitation’ included the need for careful screening for worsening symptoms indicative of Post‐Exertional Symptom Exacerbation, and the contribution of physical, cognitive, social and emotional triggers. In addition, that rehabilitation should be personalised, initially focussed on symptom stabilisation, with an awareness built into programmes that return to health is unique to each individual with LC and not necessarily linear. These recommendations built on an earlier briefing paper produced by World Physiotherapy collaborating with LC Physiotherapy in 2021 and Guidance produced by NICE in collaboration with SIGN and RCGP on the management the long‐term effects of COVID‐19. Guidance published by NICE (updated in 2024), now focusses not only on safe rehabilitation but on the value of self‐management and other forms of support.^1^, ^14^

Self‐management (support) has been an established part of many pathways of care for people living with LTCs such as diabetes, asthma arthritis and other chronic conditions^15^, ^16^ but can vary from a portfolio of techniques including information giving and education, to a fundamental change in the relationship between a patient and HCP.^17^, ^18^ While there is evidence for the effect of self‐management support on both clinical and humanistic outcomes such as confidence, skills and knowledge to live with and manage their condition, the effect is greater when support is personalised to individuals.^15^, ^18^ There is also increasing recognition of the value of personal communities in helping influence and change existing healthcare practice, and that illness management is not just an individual but a collective process.^19^ As discussed, people with LC have embraced personal online communities, which provided affirmation of their illness narratives as real and not imagined as well as support and advice to manage their symptoms and wellbeing.^20^

Self‐management programmes for people with LTCs vary widely with regard to delivery and content and those that emerged for people with LC are no different.^21^ In addition, the lack of personalisation of self‐management support can be an inevitable repercussion when HCP's experience or perceive a lack of time, patient complexity and organisational pressures.^22^, ^23^, ^24^ The particular skills required by HCP's to deliver programmes as intended, and monitoring of intervention fidelity is relatively scarce.^25^ However, programmes are more likely to be effective and sustained if they include evaluation of the fidelity and when necessary can, training, supervision and the presence of implementation champions.^24^

Building on existing evidence of successful approaches to self‐management support for people with multiple LTCs, we report on our use of co‐design methods to develop a new personalised support intervention for people living (nonhospitalised) with LC. The LISTEN intervention, for evaluation in a fully powered clinical trial. We have utilised the GUIDance for the rEporting of intervention Development framework^26^ to provide a comprehensive description of our intervention development (co‐design) activities with a view to enabling replication and future evaluation.^27^

## 2 METHODS

Our approach to intervention development was one of reframing personalised self‐management support in the context of the complexity and variability of symptoms associated with LC. This informed our decision to place narratives, experiences and concerns central to the intervention and draw on the learned and collective experiences of people living with and recovered from LC. This was supplemented by evidence from an existing self‐management approach, Bridges Self‐Management (Bridges), which was first co‐designed and evaluated in stroke but is now used extensively across the NHS.^23^, ^28^ Bridges uses resources and training co‐designed and co‐delivered with individuals living with LTC's alongside self‐management support strategies integrated into healthcare interactions and personalised to the needs of each individual.^29^, ^30^

### 2.1 Underpinning theories

A personalised approach to self‐management is contingent on the skills, language and methods used by HCPs to support individuals to share their experiences and build their own plan to manage everyday life, but also focus on priorities and activities that are most meaningful and purposeful. Bridges is theoretically informed by self‐efficacy as the most successful foundation for self‐management programmes.^31^, ^32^, ^33^ Supporting evidence shows that HCPs integrating Bridges into their care are less directive and more collaborative, facilitating individuals' problem solving, goal mastery and building self‐efficacy.^23^, ^29^

As with previous research on Bridges Self‐Management, we also drew on Normalisation Process Theory.^34^, ^35^ This was to understand how the language and techniques used by HCPs would be different to existing healthcare practice and facilitate engagement and enthusiasm as well as active and ongoing reflection during intervention delivery. By theoretically shaping our emerging key constructs, strategies and language, it also enabled the consideration of methods to evaluate adherence to intervention delivery.

We placed co‐production principles central to intervention development and this underpinned the ways in HCPs would deliver intervention sessions and the distribution of power and equity.^36^ Interactions did not presume a hierarchy of knowledge or experience, but a recognition that learning and capacity for self‐management would be positively influenced by harnessing the ideas, knowledge, and strategies of both the person living with LC and their HCP.

Drawing on the key aspects outlined above, we used the improvement methodology, Experience‐Based Co‐Design (EBCD), in an accelerated form, which has been tested and applied across multiple healthcare settings.^37^, ^38^ The detailed protocol for the EBCD activities is reported elsewhere and involved several distinct stages.^39^

### 2.2 Recruitment to co‐design stages

Two groups were invited and recruited to take part in all or some of the co‐design stages. We included people living with or recovered from LC, living in England or Wales and aged 18 years. The lack of testing available at the time meant that we included people who self‐identified as having LC defined as having symptoms persisting for longer than 12 weeks. Allied healthcare professionals (AHPs) and nurses with experience of supporting people with LC symptoms or having lived with or personal experiences (e.g., among family members or friends) were also recruited. We used a purposive sampling strategy to engage and include people with diverse backgrounds, age, gender, ethnicity and received support across the voluntary and charity sector such as LC Support and collaborated with a social enterprise, Diversity and Ability, with expertise in designing communications to reach marginalised groups. We used snowballing recruitment with support of online LC groups and the COVID‐19 Research Involvement Group managed by LC Support, and profession‐specific networks such as LC physio, OT Facebook groups.

#### 2.2.1 Stage 1: Small co‐design group meetings

We held separate online co‐design group meetings for HCPs and people living with or recovered from LC to enable both groups to share and discuss issues and experiences freely. We asked participants about their experiences of (1) living with LC, and (2) supporting those living with LC. Ahead of the events, participants were sent a ‘Welcome Pack’, with a study summary, explanation about Bridges Self‐Management and a brief itinerary with questions to guide discussions. They were also sent a self‐management resource for people with Acquired Brain Injury (ABI) co‐designed by people with ABI and their family/friends,^29^ to facilitate design ideas.

The groups included up to eight participants, usually lasted 2 h and were facilitated by people with expertise in co‐design. Groups used a virtual white board, break‐out rooms and interactive activities to generate discussion. Breaks were offered, cameras could be turned off and the pacing of meetings were determined by frequent check‐ins. For those people unable to or preferring not to take part in the group meetings, one‐to‐one meetings (via Zoom or by telephone) were offered.

#### 2.2.2 Stage 2: Mixed (large) co‐design meeting

A larger mixed co‐design group met to agree priorities for the ‘LISTEN’ intervention. Ahead of this, participants were sent summaries from the smaller groups and an outline of the areas for discussion. Participants were invited to feedback and ideas were captured contemporaneously and summarised by those not involved with facilitation. The meeting lasted 2 h. The final list of priorities from (stage 1) and break‐out rooms (stage 2) were agreed in principle at the end of the meeting. Following the meeting, a summary was circulated to all participants and they were encouraged to provide any additional thoughts via email.

#### 2.2.3 Stage 3: Generating intervention materials and content ideas

Participants from the earlier co‐design meetings formed two co‐design groups focusing on (1) training for HCPs and (2) the intervention resources. Meeting online, these participants took an active role in the layout, language and content of the self‐management resources and the content and focus of training.

#### 2.2.4 Stage 4: Narrative interviews with people living with or recovered from LC

To generate content for the LISTEN intervention resource (a book), we conducted narrative filmed interviews, lasting ∼60 min, with people living with or recovered from LC, purposively selected from previous co‐design meetings to reflect the diversity of characteristics and a variety of experiences. From these interviews, we produced vignettes and extracted video clips of specific experiences (positive and negative) for use in HCPs training, and specific extracts were used in the book.

#### 2.2.5 Stage 5: Finalising the resources, training and core principles for the LISTEN intervention

An iterative approach working with co‐design groups was used to develop the book in hard‐copy and digital form, and the training for HCPs. We collaborated with the social enterprise Diversity and Ability to ensure the layout and font size of the book was accessible and the sections easy to navigate. There were several iterative stages and drafts of the narratives and different sections of the book were sent to each of the 12 contributors. We also used Microsoft® (MS) Forms to gain feedback on decisions about the section order, section content and language used, and training for HCPs. Participants involved in previous stages of the co‐design were also invited to complete survey questions and sent a final hard copy of the new LC book, also a summary of how the intervention would be structured and training delivered. Participants who supported with the training content were invited to join one further online meeting to refine the training principles. The final intervention would be delivered as part of the Listen trial.^27^

The LISTEN project Patient and Public Involvement (PPI) group has supported the overall project design and the development of all study‐facing materials as well as communications about the study outputs. Several members have also contributed to LISTEN publications and presentations. This project has also benefitted from advice and consultation with one of the founding members of LC Support and lived experience representatives from Diversity and Ability and Bridges Self‐Management.

## 3 RESULTS

Overall, 28 people from England and Wales with lived experience of LC, and 9 HCPs living with, or supporting people with LC contributed to co‐design of the LISTEN intervention (Table 1).

**Table 1:** Co‐design participant characteristics.

	Frequency count
*People with Long Covid*	
Age	
18–25	1
26–35	6
36–45	7
46–55	8
56–65	4
66+	2
Gender	
Male	6
Female	22
Ethnicity	
White	17
Asian	1
Mixed/multiple ethnicity	5
Not reported	5
Healthcare practitioners	
Occupational therapist	2
Physiotherapist	5
Psychologist	2
Gender	
Female	9

Some participated in all stages of the process while others joined for one–off activities (see Table 2).

**Table 2:** Participants engagement in co‐design activities.

	Small co‐design meetings	1–1 discussion	Large co‐design meeting	Book meeting	Training meeting	Narrative interview	MS Form 1	MS Form 2	MS Form 3	Training refinement
P1	Y		Y		Y	Y	Y	Y	Y	Y
P2	Y		Y		Y	Y^a^	Y	Y	Y	
P3	Y		Y			Y^a^	Y	Y	Y	
P4	Y		Y	Y		Y^a^	Y	Y	Y	
P5	Y			Y					Y	
P6	Y					Y	Y	Y	Y	
P7	Y			Y		Y	Y			
P8	Y		Y	Y		Y	Y		Y	
P9	Y		Y			Y	Y	Y	Y	
P10	Y		Y				Y	Y	Y	
P11	Y		Y				Y	Y		
P12	Y		Y	Y	Y	Y	Y	Y	Y	
P13	Y		Y		Y		Y	Y	Y	
P14	Y		Y					Y	Y	
P15	Y		Y	Y		Y	Y	Y	Y	
P16	Y		Y	Y	Y	Y	Y	Y	Y	
P17			Y							
P18			Y		Y	Y^a^	Y			
P19		Y			Y	Y	Y	Y	Y	
P20		Y				Y	Y	Y	Y	
P21		Y								
P22		Y								
P23		Y								
P24		Y	Y	Y		Y	Y	Y	Y	Y
P25		Y								
P26		Y								
P27		Y								
P28		Y				Y				
HCP1	Y		Y							
HCP2	Y									
HCP3	Y		Y		Y					
HCP4	Y									
HCP5	Y			Y			Y			Y
HCP6	Y		Y		Y					Y
HCP7	Y									
HCP8	Y		Y							Y
HCP9			Y							Y

*Note*: ‘Y’ indicates participants attendance/engagement in a co‐design activity.

Abbreviations: HCP, healthcare professional; MS, Microsoft; P, participant with Long Covid.

a Six more narrative interviews were conducted after the co‐design process ended to explore themes in more detail.

**Figure 1:**
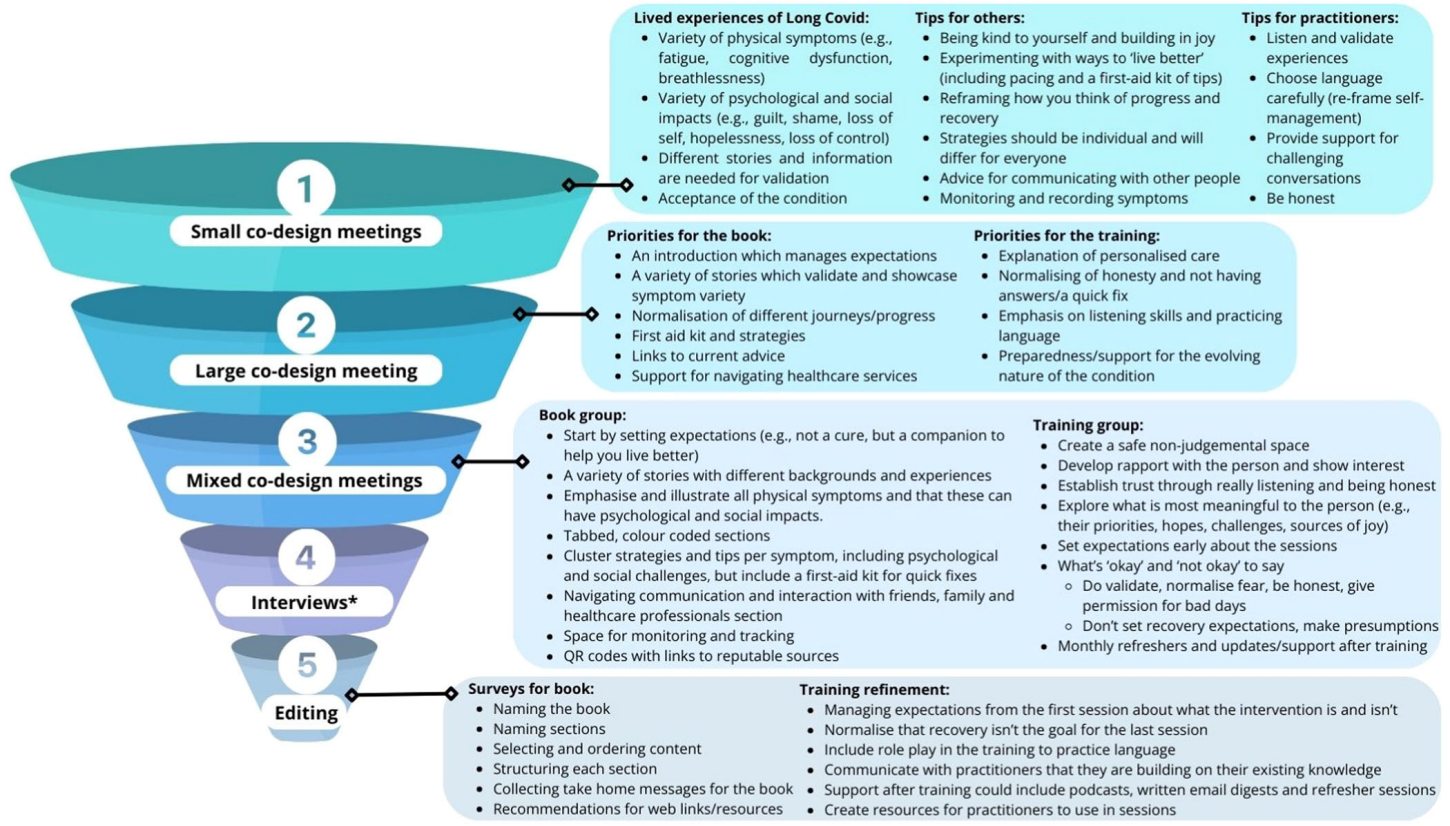
Overview of findings from each stage of the co‐design process.

The input of participants at each stage of the co‐design process is summarised in Figure 1. This illustrates the staged and iterative nature of the LISTEN intervention development.

### 3.1 Stage 1

Sixteen people with LC and eight HCPs took part in small co‐design meetings Three themes were constructed which comprised the varied lived experiences of people with LC, tips, strategies and advice for HCPs supporting care. Participants described multiple symptoms including: fatigue, breathlessness and cognitive dysfunction. These caused considerable day‐to‐day challenges and severely impacted participants' wellbeing, family, work and social activity. Impacts included fear, hopelessness, grief, guilt and a loss of sense of self. Participants discussed the importance of showing a variety of stories in any resource to illustrate the magnitude of these physical symptoms and their emotional impacts, as well as rich descriptions to support understanding.

Participants shared multiple strategies they had learnt and advice they had been given for ‘living better’ with LC. These included pacing or ‘budgeting’, accepting help from others, and advice for communicating with family, friends, work, HCPs and resting, monitoring of symptoms, being kind to yourself and building in moments of joy. One participant described how they had constructed a ‘first‐aid kit for when they had to keep going’, resembling a toolbox of easy and quick tips to use. Other participants described new things they had learnt, such as what constitutes effective rest for them. Multiple metaphors were expressed which participants felt would be helpful for others to make sense of their symptoms: for example, ‘It's like having 20‐50% charge in the phone; but making others understanding that my battery life is never going to be charge to more than 50% to start off’. Participants also shared new ways of processing the condition, including reconstruing progress and recovery (e.g., ‘we should be celebrating stability’).

Guidance for HCPs centred on the importance of listening and validating experiences. Although desperate for treatments and care services, participants described the importance of honesty even if there was nothing to offer; being told their symptoms were real and offering to stay aware of new advances in knowledge were welcomed. Participants wanted the training for HCPs to incorporate time for practicing their language and listening skills, and to avoid providing any recovery timelines. The term ‘self‐management’ was also met with some concern. With some negative connotations attached (e.g., ‘it's off‐putting because it aligns with the NHS Long Covid service of have your book and go’), participants suggested self‐management in LISTEN should be considered as enhancing knowledge, confidence, and skills to manage living with LC.

### 3.2 Stage 2

Sixteen people with LC and five HCPs took part in the large co‐design meetings, the themes were shared and discussed, and priorities agreed for the intervention resources and training for HCPs. Priorities for the book included a variety of stories which illustrate people from different backgrounds, physical symptoms, psychological and social challenges. Key content included the normalisation and validation of physical symptoms, their impact and fluctuations. These included rich relatable descriptions about relapses and nonlinear journeys illustrating that symptom relapses are not failure, but opportunities for learning. Other key priorities included the ‘first aid kit’, alongside richer descriptions of strategies that might help, links to current reputable medical and healthcare advice and support for navigating healthcare services. Finally, participants wanted an introduction to the book which set the scene and managed peoples' expectations, with a narrative of balanced hope and acceptance. In that regard, they suggested outlining what the book *is* and *is not* for, setting it within the context of LC (e.g., there are no cures, but the book is here to help you ‘live better’), and including suggestions of how things can be a little bit better, not merely the validation of difficulties.

Priorities for HCP training included an explanation of person‐centred care and language to co‐produce solutions and problem solve with people with LC. As part of this, participants suggested the need for HCPs to not have the answers, and instead work with people through trial and error to find individualised strategies. HCP participants felt not having answers could feel uncomfortable, but with a lack of medical answers this was considered a priority to normalise within training, considered as ‘being comfortable with the uncomfortable’. Time to become familiar with language and skill development was considered important to HCPs confidence to deliver the intervention. Participants agreed that language should be free of judgement, authentic and not patronising.

### 3.3 Stage 3

Following the agreement of priorities, the mixed co‐design groups focused on (1) content ideas for the book and (2) HCP training.

#### 3.3.1 Book co‐design group

Eight people with LC and one HCP took part and confirmed previous content suggestions, including an introduction setting expectations, a variety of real stories, advice for communicating with family, friends and HCPs and space for recording and monitoring symptoms. The book title was debated, and it was agreed that the title should contain ‘Long Covid’ but suggest that the knowledge base is evolving, and the end is unclear (e.g., ‘the story so far’ or ‘navigating our way through’). Content included statements such as ‘Long Covid is with us, but it doesn't define us’, and ‘it [the book] is a torch to show the path ahead, not the final destination’. Within the symptom descriptions, participants outlined the importance of emphasising that physical symptoms come first, which influence psychological and social challenges (i.e., to prevent people dismissing their LC as anxiety or a psychological condition). The unpredictability and episodic nature of symptoms was also recognised as an important message to communicate. Some symptoms were given greater priority than others to illustrate in the book, such as fatigue. Fatigue was also described through metaphors and summaries that could be read by families and friends to address some common misconceptions. For clarity, participants further recommended clustering strategies and advice per physical symptom or challenge (including psychological and social challenges) but including a generic first aid kit and an introduction to the section explaining that some tips may help more than just one symptom. Finally, QR codes and links were agreed by the groups to communicate current healthcare advice and research using accessible and relatable language that would make the information optional to engage with (e.g., ‘if you would like medical advice, in addition to hints and tips from others, please look here’).

#### 3.3.2 Training co‐design group

Seven people with LC and two HCPs took part, and several themes were constructed from participants discussion. These included the need for HCPs to create a safe nonjudgemental space during intervention sessions which would allow participants to share their story, a personalised approach to supporting people and not giving generic, impractical advice (e.g., ‘treat me like I'm my own person’) and the importance of a relationship based upon mutual trust (e.g., ‘people want to hear that we won't be dropped’). To build trust, participants expressed feeling listened to, honesty, setting of expectations (e.g., *I'm not here to cure you*) and that careful language would be vital. Language on the ‘what's ok to say’ list included ‘we are supporting you to live well’, ‘plateaus can be success as it means you're in control’, ‘it's okay to have good days and bad days’, while other generic topics included normalising fear, and giving people support to feel how they feel. Language on the ‘what's not ok to say’ list included ‘hopefully you'll feel better soon’ and ‘you should be feeling better by now’, while other aspects to avoid included personal disclosure (e.g., HCPs being back to work after a Covid infection could implicitly make people feel like a failure if they are not back to work). When considering personalised care, participants described the need for HCPs to focus on what was most meaningful at that time. For instance, some symptoms are more challenging than others, and the impact and priority for each symptom experienced should not be presumed. Instead, owing to the fluctuating nature of symptoms, HCPs should ask what is most meaningful during sessions, which may include activities that help participants to engage in small moments of joy. HCP participants suggested monthly refreshers, updates, and support beyond the initial LISTEN intervention training.

### 3.4 Stage 4

Filmed narrative interviews with 12 people with LC provided lived experiences for the book, as well as vignettes for the training. The interview data themes, published elsewhere, (see Leggat et al.) comprised three themes.^9^ First, *the landscape behind individual's LC experiences*, depicted constructions of society at the time, *such as* restrictions, case numbers and guidance. Second, the *Everyday experience of participants' LC* comprised a combination of physical, emotional and social factors, forming three subthemes: i—*centrality of physical symptoms*, ii—*navigating* ‘experts’ and *the True* ‘colour’ *of personal communities*, and iii*—rollercoaster of psychological ambiguity*. The third theme, *Personal strategies to manage everyday life* was constructed from participants' unique presentations and self‐generated solutions to manage everyday life. This comprised five subthemes: i—*seeking reassurance and knowledge*, ii—*developing greater self‐awareness through monitoring*, iii—*Trial and error of ‘safe’ ideas*, iv —*building in pleasure and comfort* and v—*Prioritising* ‘me’.

### 3.5 Stage 5

**Figure 2:**
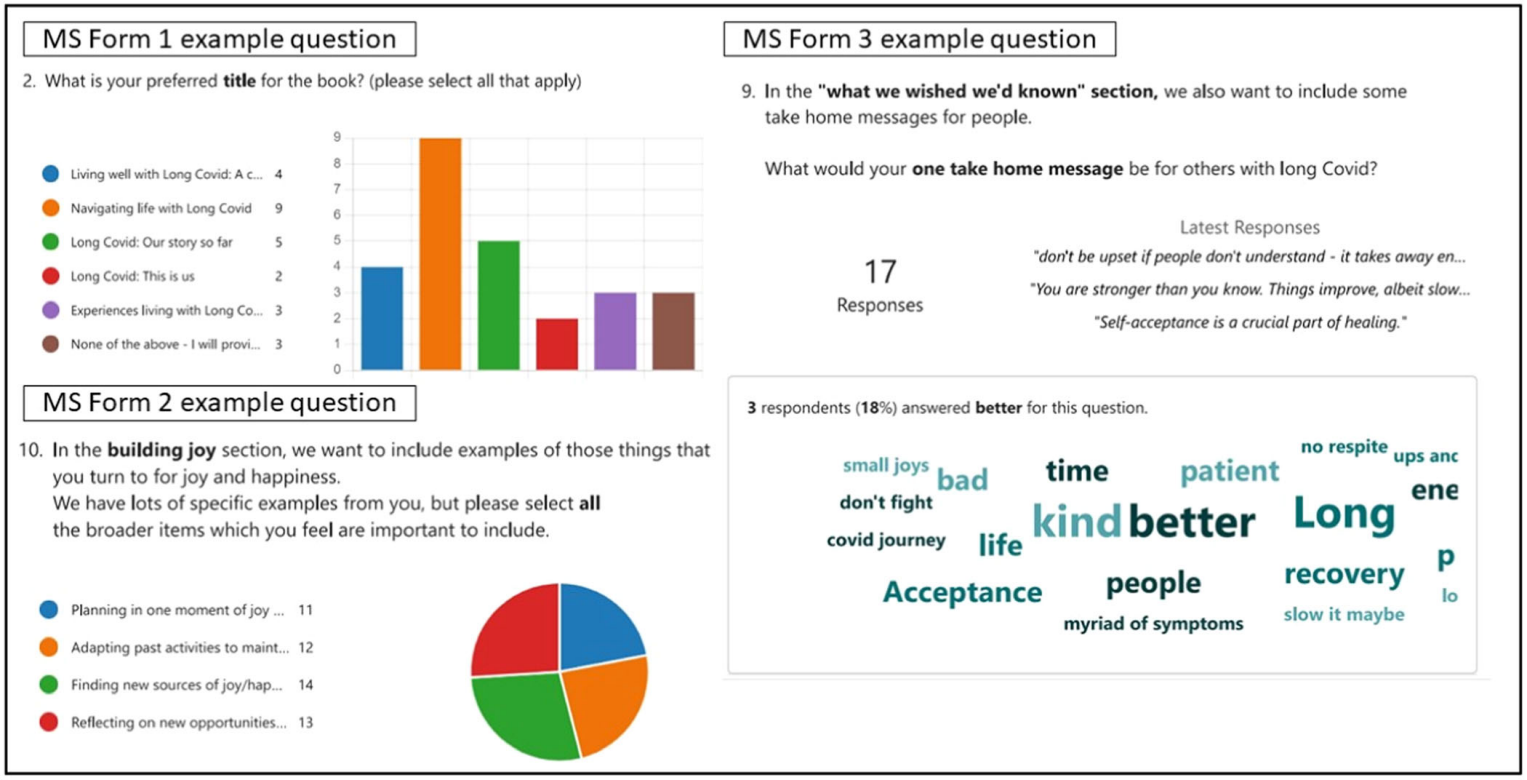
Questions and responses from the MS Forms used in Stage 5 of the co‐design process. MS, Microsoft.

The multiple‐choice questions in the MS forms survey involved up to 18 people with LC and facilitated further the decision‐making process regarding the book content. As illustrated in Figure 2, the title of the book ‘Navigating life with Long Covid: A discovery and recovery handbook’ was decided from the survey data, as were titles and the content for sections. For instance, within the ‘building joy’ section of the book, participants felt all content discussed during the co‐design process would be relevant to include, such as adapting past activities, finding new moments of joy, and reflecting on new positives and opportunities. Open‐ended questions also gathered additional rich content to use within the book sections. For instance, in the third MS forms survey, participants shared ‘one thing they wish they'd known’, which provided advice to include in the closing section of the book. All section titles and key content were chosen based upon the majority opinion.

From the training refinement meeting, components of HCP training included role play, and setting intervention expectations in the first session (e.g., not designed to fix Long Covid but support people). Co‐design members explained that the training should be pitched carefully, and not be considered a replacement to HCP's existing knowledge, but building on existing knowledge and skills gained if working in LC clinics. Resources and support following the initial training were refined and mutually agreed. These included podcasts, refresher sessions and written resources for use within participant sessions.

#### 3.5.1 The LISTEN intervention

**Figure 3:**
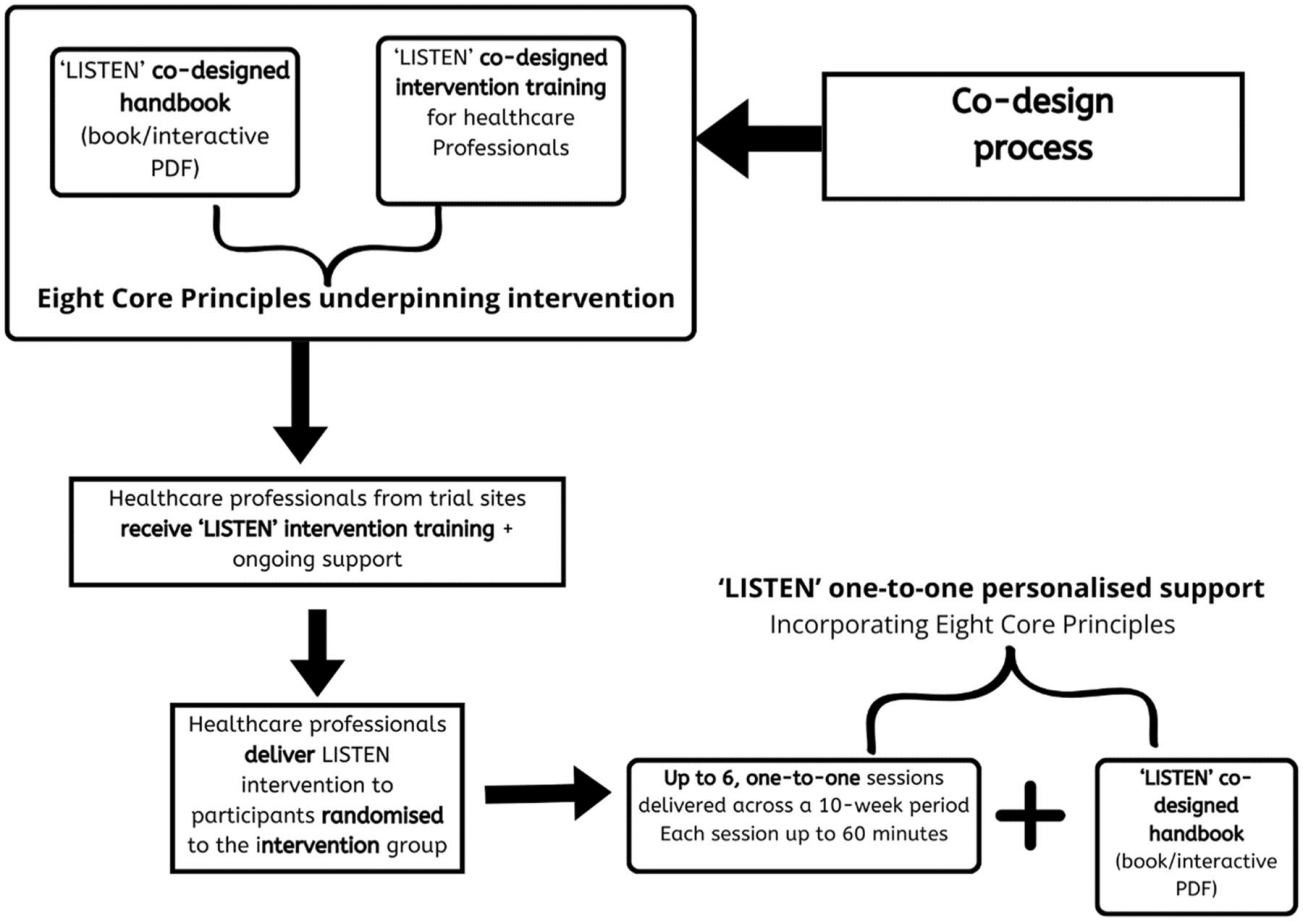
A schematic illustration of intervention development for evaluation in the LISTEN trial.

The final co‐designed intervention comprised one‐to‐one personalised support sessions. In the context of the LISTEN trial, they would be delivered via a secure web video conferencing system or telephone. Participants recruited and randomised to the LISTEN intervention could access six one‐to‐one sessions over a period of 10 weeks. Sessions were provided by HCPs who had completed at least 8 h of the co‐designed LISTEN training. The new co‐designed book would be used by them as a prompt to reinforce key discussion points within one‐to‐one sessions (narratives, ideas, solutions from other people with LC). See Figure 3 for overview.

The LISTEN book was made available to all intervention participants in accessible printed and digital, device‐friendly formats and in English and Welsh, by a professional service using forward and back translation. The book contains five main sections; Individual stories, Symptoms, Challenges and Solutions, Managing the journey, Reflect and plan and finally, a section on ‘What we wish we'd known’ (inc. weblinks). The book provides a prompt for conversations with a specific focus on learning from the LC community (their challenges, solutions, and support) and examples of daily, weekly and monthly symptom logs and appointment logs (also available digitally).

The LISTEN intervention approach drew on the construct of self‐efficacy, including mastery and vicarious experiences.^33^ The emphasis was placed on learning from the LC community and manifested through methods to reflect on small successes and learn from others in a similar position (collective evidence from co‐design participants) and trying out ideas and strategies for managing symptoms and getting to a feeling of stability and control.

HCPs delivering the LISTEN one‐to‐one sessions had to evidence experience of working with people with complex health needs across primary and secondary health settings. Core principles which were developed through co‐design stages were covered in training and integrated into one‐to‐one sessions. The core principles included:

1. Attentive listening—give time and space to individuals, validate their experiences and be present with them in every interaction.
2. Hearing beyond words, not rushing to fix—avoid prescribing fixes straight away.
3. Being curious about each individual and their story—exploration of their story/narrative to establish areas of importance and priorities, and exploration of support systems and social networks for aid beyond the intervention.
4. Exploring ways to feel more in control—reflecting on existing skills and how they can be used to problem solve and develop strategies to manage everyday challenges.
5. Using language that helps individuals experience a feeling of success from their own efforts. Supporting ways to record mastery experiences and review progress and changes over time.
6. Using language that helps individuals reflect on what has worked and their own contribution to that—highlighting that effort or maintaining a level is in itself a success, and not about achieving a goal.
7. Exploring hopes and fears as drivers for motivation—discussion and use of small steps to think ahead and navigate challenges, creating plans for working towards meaningful goals and addressing fears.
8. Alert to the possibilities of finding joy and identity—discussion of small steps to enhance individuals' new/altered/past sense of self.

A checklist was developed to evaluate whether the intervention was being delivered as intended (i.e. to establish fidelity to the core principles of the intervention). The checklist considered the language being used by the HCPs that  related to the eight core principles (see Supporting Information S1: File 1—Core Principles and Language for LISTEN intervention), plus use of the book during intervention sessions. To sustain intervention delivery fidelity, HCPs wrote notes after each session reflecting on the eight core principles. HCPs were expected to access additional learning support, available after the initial 8 h of training, which included podcasts, newsletters, exemplar sessions, frequently asked questions, guidance and crib sheets and monthly HCPs community meetings.

## 4 DISCUSSION

Using an iterative staged approach to co‐design during a global pandemic and national lockdown we have developed a new HCP delivered personalised support intervention. The core principles underpinning the intervention are embedded in the associated resources (book) and HCPs training which are assessed by adherence to fidelity. The rationale for framing the intervention as ‘personalised (self‐management) support’ was confirmed by our co‐design group but also the lack of evidence at the time to prioritise specific treatment options. National guidance promoted tailored support, information on symptoms, setting realistic goals, who to contact and accessing online resources.^40^ However, self‐management interventions predicated on education from healthcare experts and information have a weak evidence base compared to personalised models of support^15^, ^18^ and are contingent on the skills of HCPs to facilitate open and supportive conversations.^41^ Consequently for individuals navigating a new condition such as LC, which is episodic and variable,^42^ a generalised approach with an overreliance on information would have limited impact.

Our purpose throughout the LISTEN intervention development process was to reframe the approach to self‐management through comprehensive and inclusive co‐design that incorporated the views and experiences of nonhospitalised people living with LC. As such, when we referred to intervention development, we were describing the participatory processes that go beyond ‘involvement’ and recognise, as described by Palmer et al. that people with LC were ‘no longer just spectators in their care’.^43^ We were mindful in our methods that as the term co‐design has entered mainstream discourses, critics have also highlighted it is in danger of losing meaning, risking a loss of engagement when participants feel their contributions are not heard or acted upon.^44^, ^45^

The context of our intervention development was that of continued cycles of national ‘lockdown’ midway through 2021 and we navigated the difficulties in communicating with and reaching people infected by COVID‐19 and developing LC. Additionally, there were new and emerging strains of the virus, for example, delta/omicron, testing was not consistently available in England and Wales and individuals often had to rely on self‐diagnosis, with the WHO (at the time) not formally recognising LC as an ongoing condition. Politically imposed action or inaction (e.g., lockdown/mask wearing) was variable and policies and enactment of measures to stop the spread of COVID‐19 differed across the home nations (England and Wales).

During the co‐design stages, the evolution of usual care and LC services continued at pace but differed between England and Wales. In England, there was a transition to a tiered approach for the management of people with LC whereby those most affected and debilitated by their symptoms would get greater access to care. Tier 1 patients would be able to access supported self‐management either advice or through a password‐protected app such as that developed by NHS England called ‘Your Covid Recovery’, tier 2 would receive treatment in primary or community care and tier 3 access to specialised rehabilitation.^46^ In 2021, the knowledge gap and the consistency of services varied, and the aspiration for fully staffed service plans launched in the context of a crisis in the NHS workforce was challenging. This inevitably meant a need for flexibility in how services were designed and staffed, and subsequently, the numbers able to access face‐to‐face appointments were low, with virtual and online service delivery disproportionately impacting those affected by digital exclusion and poverty.^47^ By 2021, England had 89 post‐Covid assessment clinics in place and all local NHS systems had fully staffed LC service plans submitted for regions, with a view that by 2022, these services would be overseen by the new Integrated Care Systems.

In Wales, the All‐Wales Community Pathway for LC was formulated to complement the UK NICE guidelines and to provide a continuation from the existing Wales COVID‐19 Community Pathway.^48^ This was closely followed by the Adferiad Programme which sought to invest in greater LC service provision and expand community services across all health boards.^49^ By May 2023, in common with England, LC services were amalgamated with services for other LTCs.

As an intervention development team, we were also acutely aware of reports from patient‐led organisations of the inadequate access to specialist clinics across regions in England and Wales, and critically the detrimental impact of experiences of interactions with some HCPs. LISTEN co‐design participants supported these reports, which ranged from not believing that symptoms were indicative of LC or being told they would recover in the same way as flu or other conditions. We also had direct experience of individuals being told to focus on exercise and pacing despite worsening symptoms and those who in the absence of any other treatment were referred to psychological services. These experiences aligned with an early narrative study of the lived experiences of HCPs with LC published by Ladds et al.^50^ which highlighted the necessity to develop strategies such as turning towards other colleagues and others with the condition for affirmation and advice. Additionally, the impact and value to patients, of positive healthcare interactions compared to experiences of feeling dismissed and in some cases the ‘callousness’ of their experiences with healthcare systems.^50^, ^51^

While there was a clear impetus for HCPs and service pathways in the early stages of the pandemic to upskill quickly to meet the needs of a growing number of people living with varied and enduring symptoms of LC there was also a growth of ‘collective evidence’ being generated from those living with LC symptoms necessitated by the absence of support and understanding from existing health services, HCPs and society at large. This led to the exponential growth and critical role played by peer support groups such as the UK‐based peer‐led LC Support group, set up to campaign and advocate for recognition, rehabilitation, research and education to support everyone affected by LC.^52^ During this period, evidence of what works for whom and in what context came from many different directions, not least the lived and learnt experience of the people most impacted by LC.

Inequality has been a feature of LC and barriers to accessing support have been widely reported.^53^, ^54^ Unequal access to timely support personalised to individual needs could also risk exacerbating the impact on treatment outcomes and recovery. Self‐management interventions that integrate evidenced‐based theory with the real‐world lived experiences of people with LC could widen access if delivered through GP, community services or secondary care. Intervention training such as that developed for the LISTEN intervention which is interdisciplinary would also build capacity in the numbers of staff that feel equipped to deal with the complex and uncertain nature of LC while providing a trusting and collaborative relationship for those accessing services.

Overall, the LISTEN intervention has emphasised the need for contextualisation to (1) specific challenges and complexity of the condition (LC); (2) a deep understanding of the context and community setting in which the intervention is delivered; (3) contextualised training for HCPs which goes beyond initial learning and provides a community of practice to reflect, share and learn from each other and the participants receiving the intervention and (4) a focus on the adoption of specific language and techniques to support core principles of the intervention. The lived experiences of people with LC, in relation to their condition and their experiences of healthcare services have provided a primary source of evidence. The core principles embedded within the LISTEN intervention have also informed a fidelity assessment process, a key aspect of any planned evaluation.

## 5 LIMITATIONS

Data on characteristics (age, ethnicity, sex) were collected for most participants and in full for those that took part in narrative interviews (*n* = 18). However, in retrospect, we should have collected more in‐depth data on all those taking part in co‐design stages. Recruitment relied on snowballing and the goodwill of people experiencing isolation and uncertainty as well as some extreme fluctuations and complexities of living with LC. For this reason, we wanted to support a flexible approach to taking part in co‐design stages. We acknowledge this as a limitation and recognise the need to understand more about the diversity of participants including social deprivation level, education and ethnicity. In addition, females were overrepresented in both the LC and HCP groups. To some extent this reflects ONS data for LC and the gender imbalance in nursing and AHP professions.^3^, ^55^ In addition the timeframe in which to co‐design and deliver the LISTEN trial, impacted on the range of recruitment strategies to engage with community groups and ethnic health research centres.

Furthermore, all intervention development processes were conducted online. This restricted access for those people who were not comfortable with this method or not able to access meetings. We took steps to hold one‐to‐one phone calls if preferred and participants were given options to keep cameras turned off during the online meetings. We were restricted to some extent by the timeframe in which the resources and training needed to be produced. The intervention development process was completed within 8 months, and this would not have been possible without the enthusiasm and support provided by people living with the considerable impact of LC. Additionally, if the co‐design groups had not been held online, we would not have been able to reach and engage with such large numbers of participants, many of whom were housebound and experiencing energy‐limiting symptoms.

Finally, the new updated Framework for Developing and Evaluating Complex Interventions^56^ sets out the core elements that we must reflect on when progressing from intervention development to feasibility, evaluation and implementation. Complementing the framework with a middle‐range theory such as NPT has provided structure to apply the outputs from the co‐design process and build into training and ongoing support for HCPs. This will also inform the shared language and sustainable support package for HCP's ongoing learning and an implementation plan for spread across the NHS should LISTEN be found effective. Given that the LISTEN intervention has multiple interacting components, understanding of implementation determinants is critical to deliver an intervention that can be transferable and scalable. Our pragmatic randomised effectiveness and cost‐effectiveness trial in which a total of 554 nonhospitalised people with LC were randomised to either the LISTEN intervention or usual care was designed to allow us to incorporate these elements. Co‐design and PPI have been integral to the entire trial evaluation, and will facilitate the assessment of contextual influences, refinement of underpinning programme theory and will help us to address any uncertainties in relation to intervention components. Importantly this will enable us to build our understanding of what constitutes successful personalised self‐management support for people living with LC and the skills required by those delivering the intervention.

## AUTHOR CONTRIBUTIONS

**Fiona Jones**: Conceptualisation; investigation; writing—original draft; funding acquisition; methodology; validation; writing—review and editing; data curation; formal analysis; resources. **Anne Domeny**: Conceptualisation; writing—review and editing; methodology; formal analysis. **Jessica Fish**: Formal analysis; conceptualisation; funding acquisition; writing— review and editing; methodology. **Fiona Leggat**: Writing—review and editing; investigation; methodology; formal analysis; resources; project administration. **Ian Patel**: Conceptualisation; writing—review and editing; formal analysis. **Jackie McRae**: Funding acquisition; investigation; writing—review and editing. **Carol Rowe**: Conceptualisation; writing— review and editing; formal analysis. **Monica E. Busse**: Conceptualisation; investigation; funding acquisition; writing—original draft; writing—review and editing; formal analysis.

## CONFLICT OF INTEREST STATEMENT

Fiona Jones is the founder and CEO of Bridges Self‐Management a nonprofit social enterprise that was involved in the co‐design of the LISTEN intervention and training for intervention practitioners. The remaining authors declare no conflict of interest.

## ETHICS STATEMENT

Ethical approval for the intervention co‐design activities was obtained from the Kingston University Ethics Committee on 18 September 2021 (application 2887) and for the LISTEN trial from Committee (REC) For Wales (Wales REC 7), recognised by the UK Ethics Committee Authority, REC reference 21/WA/0368 on 13 December 2021. Informed consent to take part in the co‐design groups and narrative interviews was sought before participation and participants were provided with the right to withdraw from the study at any point without giving a reason. Informed consent was retrieved from all participants in the study. Consent forms were sent via email with participants signing electronically and returning the forms through email. All consent forms are stored securely in a folder in a university password‐protected drive accessible only to the research team and will be destroyed after a period of 10 years. All procedures were followed in accordance with the Declaration of Helsinki.

## Supporting information

Supporting information.

## Data Availability

The data that support the findings of this study are available on request from the corresponding author. The data are not publicly available due to privacy or ethical restrictions.
