# Supplementary material for: Using co‐design methods to develop new personalised support for people living with Long Covid: The ‘LISTEN’ intervention
Source: Health Expect. 2024 May 24;27(3):e14093. doi: 10.1111/hex.14093 (PMC11116943; doi:10.1111/hex.14093)
Supplement: Supplementary file 1 — Supporting information. [file HEX-27-e14093-s001.pdf]

LISTEN Key Phrase Diagram

Green - skills you should use across all intervention sessions
Light blue – skills you may use more in the earlier/middle sessions
Dark blue – skills you may use more in the middle/later sessions

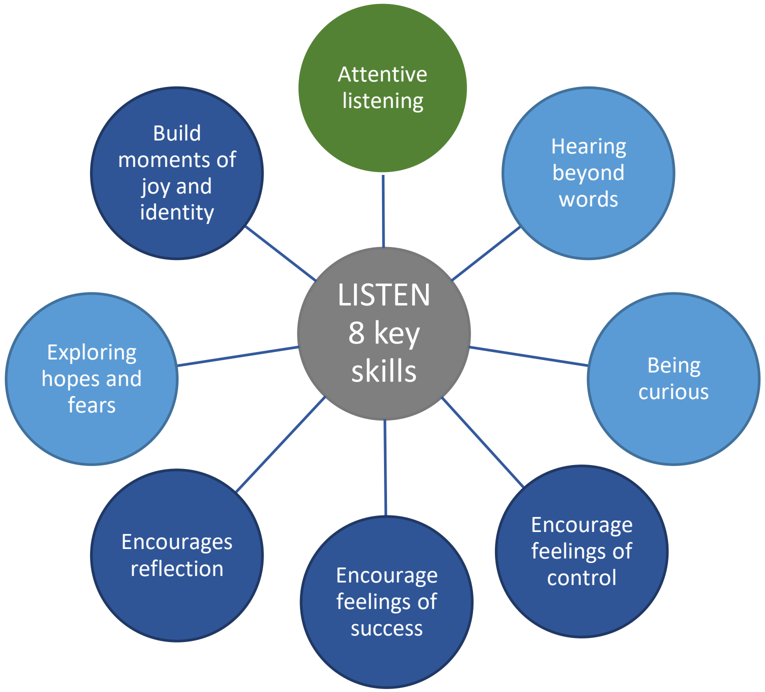

Being alive to the possibilities of finding joy and a new identity after long Covid

- Why did you enjoy doing the hobbies/interests you did before?
- What might you be able to do now to experience a similar feeling?
- What's one small thing that would make you feel like yourself/moving forward? How can you include that in your day?
- What can you build into each day that will bring some happiness?
- What time of day can you try to do something you enjoy?

Exploring hopes and fears as drivers for motivation

- What do you care most about right now?
- What's most important to you going forward?
- What would success look like to you?
- What's one thing that would make you feel like your old self?
- What's the worst that could happen?
- What's stopping you?
- What would happen if you tried it like this?

Using language that helps individuals reflect on what has worked and their own contribution to their success

- How did you do it? How did that make you feel?
- What felt wrong/right about that?
- Why do you think you felt that way?
- How do you think you did?
- What changes have you noticed? Why do you think they might have happened?
- How confident do you feel about …?
- What affect is … having on you?

- What advice would you give to others?
- What will you take forward?
- What ideas might you use/not use again?

Using language that explores how individuals can get a feeling of success from their efforts

- What's one small step towards that?
- What can you do that will get you closer to that?
- How could you move yourself from a 5 to a 10 on confidence scale?
- What have you learnt before that you could try here?

- What have you been able to try since I last saw you?
- What have you learnt about yourself here?
- What are you pleased about?
- What would you like to be doing more of or try doing?
- How might you like to record that?
- It might help to have a reminder of that…

Hearing beyond words and not rushing to fix

- What are you having difficulties with now?
- Tell me more about that…
- What does that feel like to you?
- What has worked for you so far?
- It's okay to feel that way…
- That must be challenging…

Being curious about individuals and their story

- What were/are your interests/hobbies?
- What things are important to you?
- What things make you feel more like yourself?
- What was your usual daily routine?

- What have you had difficulties with?
- What's your understanding of…?
- What do you feel triggers this?

- What do you know about the things available?
- What information do you like looking at?
- What have you used already?

- What is most important to you right now?
- What do you care most about right now?

Exploring how to help individuals feel more in control

- What has worked well before? How did that feel?
- How would you normally tackle this?
- How could you remember ways of managing this, what works for you as a prompt?

- What has worked for others that you could try?
- How can we adapt this to make it practical for you?
- What's one thing you could try this week?
- What ideas would you like to experiment with?
- What would help you feel a little more in control?
- What could you try if you have a setback?
- Who or what could help you with this in the future?
- What support might you need if this happens again?
- What could you plan to use again?
